# Supplementary material for: Are carbon emissions trading and green financial instruments synergistic? -Comprehensive quantitative research based on content analysis
Source: PLoS One. 2024 Mar 7;19(3):e0298601. doi: 10.1371/journal.pone.0298601 (PMC10919601; doi:10.1371/journal.pone.0298601)
Supplement: S1 Data — (ZIP) [file pone.0298601.s001.zip › Data/地级市Z_F工作报告.zip!/║┌┴·╜¡/║╫╕┌╩╨/└┤╫╘╣1⁄2╓┌║┼í╛┬φ┐╦╩2╛▌═°í┐_2018.docx]

各位代表：

　　现在，我代表市人民政府，向大会报告工作，请予审议。并请各位政协委员和其他列席人员提出意见。

　　一、2017年工作回顾

　　2017年是本届政府开局之年。面对复杂多变的形势、经济下行的压力和繁重紧迫的任务，我们在省委、省政府和市委的正确领导下，在市人大、市政协的监督支持下，紧紧团结和依靠全市人民，牢牢把握大局，妥善应对挑战，团结一致，顽强拼搏，全市经济实现逆势增长，社会事业协调发展，圆满完成了市十六届人大一次会议确定的目标任务。预计全年实现地区生产总值同比增长7%、规上工业增加值增长15%、固定资产投资增长7%、社会消费品零售总额增长9%、公共财政收入增长14.7%、外贸进出口总值增长91%、城乡居民收入分别增长6.5%和7%以上。

　　——过去一年，我们多措并举协调推进，着力优化产业结构，经济发展增势强劲，综合实力明显提升。一是农业经济全面发展。粮食总产量突破25亿斤。农民专业合作社、家庭农场数量稳步增长。建成“互联网+农业”高标准示范基地46个，有机食品标志达到9个、绿色食品标志达到67个。绥滨县被评为“三产融合”试点县、省级农产品质量安全示范县，萝北县被评为国家级水稻高产高效示范基地、国家级农产品质量安全示范县。二是工业经济稳中向好。煤炭行业作为传统支柱产业作用明显，龙煤鹤矿生产原煤保持在1000万吨左右，实现税收3.28亿元；预计地方煤矿生产原煤400万吨，同比增长124%，利税同比实现翻番。涉煤行业发展势头良好。预计全市规上工业完成增加值65亿元，同比增长15%。预计非公经济完成增加值84.7亿元，同比增长16.5%。三是第三产业繁荣活跃。“好品鹤岗”线上商城正式上线，电商产业园、京东“互联网+新经济”基地等项目投入运营。绥滨县成为第一批省级电子商务示范县。金融机构各项存、贷款余额分别增加39.1亿元、10.4亿元。旅游业发展快速，预计全年接待游客和旅游收入分别增长20%和30%。建立了沿黑龙江旅游产业带、广东“2+4”等合作联盟。成功举办鹤岗旅游产品北京推介会，与首旅集团签订了合作框架协议。大力发展外贸物流产业，与俄方就开展跨境电商、建设跨境公路大桥、俄罗斯商品集散中心等事宜达成深度共识。萝北互市贸易区建成开通，赴俄农业种植面积超过2万公顷。

　　——过去一年，我们立足实际谋划长远，着力抓好招商引资，项目建设步伐加快，支撑作用显著增强。一是产业项目加快发展。累计实施千万元以上产业项目136个，亿元以上项目45个，完成投资28.84亿元。加快发展绿色食品产业。东方希望集团生猪产业项目开工建设，米家乐万吨绿色方便米饭、盛中农业大豆分离蛋白等重点项目加快推进，可口儿大豆产业园一期工程、新龙跃胚芽米深加工、银沐生物科技食用菌、金鹤1万吨精酿啤酒和2万吨纯净水等5个亿元项目建成投产。加快发展煤化工产业。中海油华鹤10万吨乙二醇项目完成可研编制，世纪云天化BB肥、北京三聚环保炭基复合肥项目稳步推进，睿泽科技百万吨乙二醇项目前期工作有序开展。加快发展石墨新材料产业。推进石墨新材料产业基地建设，华升石墨烯、帝源石墨、奥星石墨负极材料等项目进展顺利。加快发展战略新兴产业。国信通动力电池、手机和负极材料项目开工建设，京东鹤岗地方体验馆等项目成功落地，与中国中药控股公司合作建设的“中国中药鹤岗产业园”项目正式签约。二是招商引资力度加大。先后赴北上广深等地开展外出招商和项目洽谈活动16次，组织参加了第29届哈洽会、第5届黑龙江绿博会等经贸活动20多项。三是园区建设基础夯实。高起点编制园区规划，健全工作机构，加大园区基础设施建设力度，园区各项工作步入正轨。四是重大建设取得进展。鹤佳快速铁路项目纳入《省交通运输体系发展“十三五”规划》；鹤岗民用机场项目通过国家民航局、北部战区空军选址审批；绥名公路改扩建、鹤大高速鹤岗至佳木斯段改造开工建设，采取PPP模式运作的南岗路、鹤立湖景区旅游公路、鹤大公路与哈肇公路连接线建成通车；名山港码头改扩建、中兴港建设进一步加快；关门嘴子水库通过国家发改委技术评估。

　　——过去一年，我们始终奉行民生至上，着力推进惠民实事，民生建设成果丰硕，社会事业蓬勃发展。就业形势持续稳定。新增就业2.3万人，城镇登记失业率控制在4.1%以内。举办专场招聘会47场，为385名创业人员发放贷款2000万元，有效促进科技人员、高校毕业生、龙煤分流人员、贫困人口等群体实现就业。保障水平持续提升。持续扩大“五险”覆盖面，参险人员新增2.9万人，最低工资、养老金、城乡低保均有提升，社区干部工资提高500元以上。积极开展救助行动，筹集资金2亿元，对各类困难群体进行救助。安居工程持续推进。各类保障性安居工程共开复工建设4万套，安置居民2.6万户，完成货币直接补偿9277户，办理不动产权证2万户，改造农村危房703户。社会事业持续进步。扎实推进脱贫攻坚，帮助贫困村落实各类产业项目49个，整村推进了22个贫困村的“三通三有”基础设施建设。不动产登记制度改革走在全省前列，启动了历史遗留项目不动产权证办理工作。三个行政区通过义务教育均衡发展国家验收，8所幼儿园晋升为省级示范园，鹤岗一中被评为省级高中改革发展示范校，鹤岗职业技术学校组建完毕。萝北县成为国家级公立医院综合改革示范县。全市标准化村卫生室建设规范化率达100%。开展大型群众文化活动演出32场，乡镇综合文化站实现全覆盖，22个农村文化广场全面落成，100个“互联网+专网”数字图书馆终端全面建成。广泛开展全民健身运动，加强群众身边体育设施建设。成功举办“泛舟龙江”、“劲道杯”轮滑公开赛等系列活动。创新创业持续发展。与北京中关村孵化中心和哈尔滨中关村孵化基地合作，打造了省内首家政府主导的“双创”平台和孵化器航母；3家科技企业孵化器、2家众创空间建设完成，双创工作成效显著。社会治理持续加强。大力推动安全责任体系建设，全面落实安全生产“四个责任”，各类生产安全事故起数下降11%，全年无重大及以上事故。地方煤矿整治整合关闭工作稳步推进。认真落实信访工作责任制、信访接待日和重点案件领导包案制度，有效化解各类信访积案。持续开展严厉打击黄赌毒、黑拐骗犯罪专项整治行动，巡逻防控更加严密，刑事案件发案起数下降16%，“天眼工程”实现重点部位全覆盖和无缝对接，全市保持安定和谐的良好局面。

　　——过去一年，我们扎实推进“城市双修”，着力完善城市功能，城建力度前所未有，城市面貌焕然一新。一是加快推进城市功能修补。今年是城市建设投入最大、项目最多、速度最快、成效最明显的一年。突出规划引领，推动多规合一，《鹤岗市城市总体规划》获得国务院批复。重点实施了市人民剧院、体育馆升级改造工程。新、改建主次道路65条，第三跨线桥、城市西外环公路开工建设，城市中轴线道路建设改造工程实现了当年立项、当年建设、当年竣工，道路沿线通讯、给水、供热等基础设施配套工程同步施工。更新、亮化了34条主次道路，方便了百姓出行。启动了老旧小区改造工程，改造405栋、2.4万户住宅楼。改造供热和供水老旧管线28.7公里和14公里，新建了东山区兴华村段的供水管线，解决了困扰900余户居民和30余户园区企业多年的用水困难问题。建设了30座水冲公厕、3座垃圾中转站和5处移动式垃圾压缩站，垃圾清运机械化率达到85%。新建了10处停车场，新增停车泊位786个。深入推进“两城”创建工作，集中开展了城乡环境综合整治。二是加快推进生态环境修复。淘汰建成区燃煤小锅炉228台，拆除110座废旧烟囱，空气质量达到国家二级标准超过300天。扎实推进了噪声治理和医疗废物处理等工作。加大市域内秸秆焚烧管控力度，全力降低污染物排放。全民义务植树68万株，打造了30公顷南风井景观带和15.4公顷南山路景观带，升级了天水湖绿地、五指山公园，新建主次干道绿地8处。城市饮用水源水质达标率100%。三是加快推进绿色矿山综合整治。建立健全矿山生态环境和地质灾害监测体系，推进煤矿边坡治理、尾矿治理、采空区治理、土壤改良、植物修复和水资源修复。开展废弃矿山治理，整治矿区废弃地、关闭矿井、地面塌陷坑42万平方米。

　　——过去一年，我们提高效率转变作风，着力抓好自身建设，履职能力全面强化，行政效能日益提升。政府系统各部门认真学习贯彻党的十九大精神和习近平新时代中国特色社会主义思想，大力弘扬“认真、实干、创新”的新时期鹤岗精神，深入开展“两学一做”、“学走改促”活动，各级干部作风持续转变，干事创业的精气神明显提升。扎实推进“放管服”改革，积极推动政府事项流程再造。严格遵守重大行政决策法定程序，不断完善依法科学民主决策机制。主动听取社会各界意见和建议，自觉接受人大法律监督、政协民主监督和社会舆论监督，全年共办理人大代表建议87件，政协委员提案128件。全面落实党风廉政责任制，廉洁政府建设取得新成效。大力推进政务公开，政府工作更加规范、透明。

　　统计、物价、外侨、老龄、双拥、人防、地震、气象、档案、民兵、预备役、关心下一代等工作取得新的进展，民族宗教、妇女儿童、残疾人、红十字等事业取得新的进步。

　　各位代表，2017年政府各项工作在困境中扎实推进，扭转了经济下滑势头，办成了一批民生实事，维护了社会和谐稳定，成绩来之不易。这些成绩的取得，是省委、省政府和市委统揽全局、正确领导的结果，是市人大、市政协有力监督、大力支持的结果，是全市各级、各部门破解难题、苦干实干的结果，更是全市人民团结一心、努力奋斗的结果。在此，我代表市政府，向全市人民，向各民主党派、工商联、无党派人士、人民团体和社会各界人士，向中省直各单位，向驻军指战员、武警部队官兵，表示衷心的感谢！向奋战在生产工作第一线的煤矿职工、环卫工人和公安干警，向各行各业辛勤工作的广大干部职工表示崇高的敬意！

　　在总结成绩的同时，我们清醒地看到前进中还存在一些困难和挑战。我市经济运行仍处于不确定状态，经济结构不优、产业培育质量不高、企业竞争力不强、财政支撑不够的问题仍很突出，发展的内生动力严重不足；民生改善任重道远，就业压力依然较大、人居环境尚需改善、社会保障任务繁重、社会事业发展滞后、社会治理还有差距；城市基础设施建设欠账较多。另外，年初安排的工作，还有个别工程项目因特殊原因没有完成；在一些重点工作的推进中，还有一些群众不满意的地方。我们要进一步增强发展的责任感和紧迫感，采取切实有效措施，认真加以解决，不辜负全市人民和各位代表的期望和重托。

　　二、2018年工作安排

　　2018年是贯彻党的十九大精神的开局之年，是决胜全面建成小康社会的关键一年。刚刚闭幕的市委十二届二次全会深入贯彻落实党的十九大精神和省委十二届二次全会精神，在全面总结过去一年工作、科学分析当前和今后一个时期形势的基础上，绘就了未来工作的宏伟蓝图。我们要坚定发展信心，把握发展大势，落实好市委提出的“9+1战略”，把党的十九大精神和省、市委全会精神转化为鹤岗转型发展的生动实践。

　　2018年政府工作的总体要求是：深入贯彻落实党的十九大精神，坚持以习近平新时代中国特色社会主义思想为指导，坚定不移践行新发展理念，牢固树立以人民为中心的发展思想，坚持稳中求进工作总基调，坚持质量第一、效益优先，以供给侧结构性改革为主线，推动经济发展质量变革、效率变革、动力变革，着力实施“四大战略”，培育发展“五大产业”，大力推进“9+1战略”，更加注重全面深化改革、现代产业体系、保障改善民生、城市建设管理、生态环境保护、社会治理创新和政府自身建设，努力打造“三地一区”，加快建设生态强市、旅游强市、工业强市、农业强市，为加速转型发展、决胜全面小康，建设富强民主文明和谐美丽的社会主义现代化新鹤岗而努力奋斗！

　　2018年全市经济社会发展的主要预期目标是：地区生产总值增长7%以上；规模以上工业增加值增长10%；固定资产投资增长10%；公共财政收入增长10%；社会消费品零售总额增长10%；外贸进出口总值增长10%；城镇常住居民可支配收入和农村常住居民可支配收入分别增长7%以上。

　　依据上述总体要求和目标，重点做好五个方面工作：

　　(一)大力发展“五大产业”，重点打造“三地一区”

　　要坚持把发展作为解决一切问题的关键，认真贯彻中央关于“高质量发展”的要求，把产业项目建设、创新驱动与新增长领域培育紧密结合，大力发展“五大产业”，重点打造“三地一区”，坚决打好转方式调结构攻坚战。

　　一要大力发展绿色矿业，重点打造“煤头化尾”示范基地。充分挖掘我市资源优势，创新矿产资源开发模式，培育矿业经济发展新增长点。对优势矿产资源进行勘查、开发和利用，提高政府对资源的掌控力度和优化配置能力，逐步实现资源优势向产业优势转变。稳定煤炭生产，支持龙煤鹤矿集团稳产增效；推进地方煤矿整治整合，把煤矿集团做实做强，提升煤矿机械化、信息化、自动化水平，建设本质安全型矿井，确保地煤产量稳定在500万吨以上；推进中海油华鹤煤化工新华矿井及选煤厂项目。加快传统煤化工产业转型升级，支持中海油华鹤、征楠煤化工等现有企业稳产提效，加快实现煤炭从基础化工产品向高端化工产品、从生产粗放型向资源节约型转变。促进产业精深发展，重点发展煤制焦化及其副产品深加工、煤制肥及高效复合肥和尿素下游产品深加工、煤制乙二醇等3大产业链条。力争用三年时间，初步建成“煤头化尾”示范基地。

　　二要大力发展生态农业，重点打造“两江一岭”绿色食品加工基地。抓好农业生产，调优农业结构。引导发展杂粮杂豆、鲜食玉米、汉麻等特色种植。大力发展畜牧业，重点推进东方希望集团300万头生猪和绥滨肉鹅产业化项目。加快发展林下经济。增加绿色农产品供给，做优做精做强绿色有机种植业。打造农产品知名品牌，积极创建国家级出口食品农产品质量安全示范区。加快农业基础设施建设，全力打造绥滨优质水稻主产区、萝北杂粮杂豆特色区、市郊蔬菜基地保护区；建设农业物联网试验示范基地。以“粮头食尾”、“农头工尾”为引领，推进农业产业化。支持绥粮集团做大做强，加快米业企业整合与升级改造。发展精细农产品加工，全力推进可口儿大豆食品产业园、米家乐方便米饭等重点项目。强化农产品营销，提升农产品流通现代化水平，建成南翔农副产品批发加工市场，推进万圃源蔬菜批发市场农产品冷链物流建设；扶持涉农电商企业做大做强，推动农产品线上线下互动发展。促进农民增收，发展农民劳务产业。力争用三年时间，把我市农林食品产业打造成支柱产业。

　　三要大力发展文化旅游产业，重点打造“龙江三峡”中俄犹文化旅游集合区。创新旅游开发模式，坚持“界江游、矿山游、现代大农业游、森林游”四个重点发展方向，加大与北京首旅集团等战略投资者合作力度。加强旅游与文化、教育、养老等跨界融合，打造时尚旅游产品。借助国内外大型展会营销推介旅游产品。深化与广东"2+4"等区域旅游联盟合作，互推线路，拓展市场。加快旅游景点建设，抓好“一江两岛三峡”设施完善；全力推进谕霖射击场二期、鹤立湖景区二期、清源湖景区二期等重点工程；支持鹤岗矿史馆、天水湖冰雪乐园、凤凰山滑雪场扩展旅游项目；谋划推进狩猎场项目。积极发展乡村旅游观光、农耕体验、田园采摘、绿色餐饮、休闲度假等新业态。加强旅游基础设施建设，完善景区游客接待中心、旅游集散中心、水冲公厕等服务设施；加快林区旅游道路建设，开通重点景区季节性旅游公交线路。

　　四要大力发展战略新兴产业，重点打造石墨新材料产业基地。成立石墨产业推进办公室，创建石墨产业发展联盟，建设石墨工程及院士专家工作站和国家石墨检测中心。全面对接国家和省相关战略规划，合理规划石墨产业布局，加快建设萝北云山石墨基础材料、鹤岗石墨新材料两大产业园区，积极引进一批关联度大、产业链长的龙头项目，培育壮大一批具有核心竞争力的生产研发企业，力争用三年时间把鹤岗打造成引领中国石墨新材料产业发展的集聚示范区和具有全球竞争力的石墨新材料产业化基地。完成国信通智能手机和动力电池一期建设并投产，推进二期工程启动，开工建设石墨采选项目。全力抓好中铁云山等石墨勘查项目“探转采”，确保矿山开采和深加工项目按期实施。推进华升、奥星、帝源高纯石墨、负极材料等项目。在其他战略新兴产业发展上要有新突破。发展新医药产业，推动双兰星制药公司与中国中药集团深入合作。发展新能源产业，重点推进和禾生物质发电、天合集团光伏发电等项目。发展新一代信息技术产业，重点推进康仕电子科技、云基地、城市WiFi等项目，谋划建设智慧城市。发展现代商贸产业，加强对比优特、喜家德总部经济扶持力度；加快实体零售创新转型步伐，支持宏益家电等企业申报省级实体零售创新转型试点企业。

　　五要大力发展外贸物流产业，重点加大对外经济技术合作。全面加强与俄犹太自治州、比罗比詹市、哈巴罗夫斯克市等政府和企业层面的交流与合作，扩大与韩国及港澳台地区的经贸合作，进一步提高对外开放水平。加快发展进出口贸易，抓好五矿萝北、黑金石墨、北方石墨、清华紫光等企业外贸进出口运行；大力培育新的外贸主体，进一步扩大大豆、木材、俄罗斯商品进口和建材、机电产品出口规模。深入开展对外经济技术合作，全力推进赴俄种植养殖、赴俄锰矿开采等重点项目。建成鹤岗俄罗斯生活体验中心。推进跨境基础设施建设，全力打造名山口岸沿边重点开发开放试验区，多措并举推动萝北中俄边民互市贸易区发展壮大。支持南翔国际物流园区国检试验区和海关监管库建设，带动跨境物流行业快速发展。

　　各位代表，“三地一区”是我市今后的发展目标，“五大产业”是我市转型发展的支撑。我们要坚持转方式、调结构，加快构建现代产业新体系，努力实现更高质量、更有效率、更可持续的发展，建设工业强市和农业强市！

　　(二)加强基础设施建设，提升城市承载能力

　　启动“城乡建设品质提升年”活动，坚持高起点规划、高质量建设，实用性与艺术性相融合，建设一批精品工程，全面提升城市功能品质，让人民群众生活的更美好、更舒心。

　　一要坚决打好重大基础设施网络建设攻坚战。全面开展鹤佳快速铁路项目各项前期准备工作，确保明年开工建设，2020年建成通车，实现市民快速出行、舒适出行、方便出行。加快推进鹤岗机场筹建工作，确保明年一季度获得国务院、中央军委立项批复，年底前开工建设，2020年建成通航；推进通用机场建设工作。新建、续建、谋划公路交通项目19个。重点完成绥名公路、国防路等续建项目，开工建设机场路、哈肇路、旅游公路等重点工程，积极谋划鹤伊高速公路项目。加快建设绥滨中兴港，打通新的口岸通道，全力谋划中俄名山黑龙江大桥项目。加快推进关门嘴子水库前期工作，力争明年获得国家批复开工。

　　二要实施城市路网升级工程。坚持城市道路与小区配套道路无缝衔接，进一步解决破损路、断头路、瓶颈路等影响群众生产生活问题。升级改造铁西路、南选路、工业街、学府路、财富路、兴山路等9条城区道路，全力推进经一路、纬五路建设，贯通西部环线，确保棚改项目小区外道路全线开工建设。实现第三跨线桥竣工通车。在红旗路人民办事中心地段建设一座高标准人行过街天桥。坚持管网改建与道路建设同步推进，改造“跑冒滴漏”老旧供热管网27.2公里，实施供水老化管网和水表出户改造工程。

　　三要加快公共服务设施建设。全力推进华能电厂市区供热工程，彻底解决市区小锅炉供热过多、供热质量不高的问题，保障冬季百姓住上暖屋子。抓好南奋斗路等新建道路路灯亮化；改造提升财富路、兴山路等老旧路灯；健全延伸化工街、砖厂街等背街背巷路灯设施。新建一批小型停车场和停车位，缓解城区“停车难”的问题。配齐新型环卫设施，新建5座移动式垃圾转运站，新建一批水冲式公厕，在主城区消除旱厕，努力补齐影响群众生活品质的短板。启动建设“五位一体”综合工作训练作战基地，完成体育馆改造工程。完善民政基础设施，翻建五公里殡仪馆，筹建新华第二殡仪服务中心。统筹抓好细鳞河水库除险加固、北山净水厂移址重建、农村供排水等重大工程，让百姓喝上放心水。

　　各位代表，城市是经济社会发展和人民生产生活的重要载体，是现代文明的标志。我们要不断完善城市功能、提升城市品位，努力让全市人民生活的更加美好幸福！

　　(三)加快生态系统修复，努力建设美丽家园

　　实施“生态立市”战略，坚持生态建设优先于城市建设的理念，以创建旅游城市的标准推动生态建设与城市建设相融合，同步规划，互为支撑。要合理划分生态保护区、生态整治区和重点建设区，增强生态系统修复建设的科学性和实效性，加快生态强市、旅游强市建设。

　　一要坚决打好污染防治攻坚战。启动“鹤岗蓝”三年保卫战，强力推进秸秆禁烧及综合利用，统筹抓好小锅炉撤并、清洁改造和尾气监管，杜绝间歇性重污染天气。实施治污净水行动，按照“总体规划、分步实施”的原则，推进“两河十四沟”清水秀岸综合治理工程，年内完成鹤立河水位提升工程，前进沟天水湖南门至欣虹桥，石头河起点至南翼桥，黎明沟、老头沟、头道沟、二道沟、龙须沟等综合治理，将每一条水系都建成带状公园，实现水清岸秀。全面落实“河湖长制”要求，制定好水源地保护条例。加大矿山环境治理力度，全面整顿主城区周边非煤矿山，对破坏道路、污染水源、影响群众生活的打石场、采沙场坚决关停。加快推进城区煤场整合，组建4个煤炭集中交易市场，提高运输防护标准；坚决打击清除私开私建的小煤场。在全面整治的同时，鼓励引导社会资本利用废弃矿山发展旅游、休闲、观光、农林产业。开展矿区绿化硬化行动，启动岭北露天矿地质环境综合治理。

　　二要着力加快森林城市创建。坚持城区林区协同发展，力争用三年时间建设成为国家森林城市和省级生态市。建立最严格的森林资源监管制度，严厉打击毁林开垦和非法侵占林地等违法犯罪行为。突出我市山水相映、林水相依的特色，加快建设城市北郊森林公园、十里河国家级湿地公园、大砬子山森林公园、大架山花果观赏园、南翼森林植物园、五指山公园、麓林山风景区公园。提高城市绿化美化标准，完善主次干道两侧景观绿化，打造城市各出口绿化景观带。鼓励社会资本投入城市绿化，引导市民参与栽花种草植绿行动，用绿色美景装扮学校机关企业、街道社区院落，把鹤岗打造成群山环绕、水清岸秀、城在林中、人在景中的绿色生态之城，让百姓享受人与自然和谐共生的高品质生活。

　　三要坚决打好活力边城、美丽乡村建设攻坚战。开展农村人居环境整治三年行动，持续加大农村环境整治力度。以抓好东山日新村、前进村垃圾治理、“厕所革命”为重点，打造示范样板，形成可复制的经验，推进9个城郊村改造，让农民享受城市便利设施、让市民体验农村田园生活，形成良性互动，推动城乡融合发展，以此为突破口带动全市美丽乡村建设。紧紧围绕“六美”要求，加快推进绥滨集贤村、萝北金沙村、兴安峻德村等10个示范村和2个示范片区建设，全力打造一批产业兴旺、生态宜居、乡风文明、治理有效、生活富裕的现代化美丽乡村。

　　各位代表，建设美丽家园是全市人民的共同愿望。我们要牢固树立社会主义生态文明观，着力保护好我们的“山水林田湖草”，还鹤岗大地青山绿草、碧水蓝天！

　　(四)做好保障和改善民生工作，不断增强人民群众获得感

　　保障和改善民生没有终点，只有连续不断的新起点。要抓住百姓最关心最直接最现实的利益问题，把握重点、节奏和可持续性，既尽力而为，又量力而行；要坚守底线、引导预期，既不降低标准，也不吊高胃口，一件事情接着一件事情办，一年接着一年干，持之以恒把民生工作抓实抓好。

　　一要坚决打好精准脱贫攻坚战。坚持精准扶贫、精准脱贫，加大产业、金融、社会保障等扶贫力度，确保按期实现“县摘帽、村出列、户脱贫”目标，决不让一个贫困群众在全面建成小康社会的进程中掉队。同时，要高度重视并切实抓好城区贫困人口脱贫解困工作。

　　二要着力促进更高质量就业。就业是最大的民生，要实施就业优先战略，促进经济增长与扩大就业联动，支持快递、物流、电商、微商等新就业形态发展，发挥小微企业就业主渠道作用，发展创业载体，以创业带动就业。要开展就业服务专项活动和就业技能培训，持续推进科技人员、大学生、农民、城镇转移就业职工等重点群体创新创业。落实创业担保贷款、“援企稳岗”、灵活社保补贴等政策，用好就业补贴资金。2018年新增就业2万人，城镇登记失业率控制在4%以内，确保“零就业”家庭动态清零。

　　三要着力建设良好人居环境。加快推进保障性安居工程建设，把生态环境好、升值空间大的地段用于建设保障房，解决好老城区、主城区棚改遗留问题。完善已建成棚改安居小区的管网、绿化、亮化、文化、体育、公交、菜场等生活服务设施，提高居民生活便利性。引导小微企业进入小区，发展生活性服务业。支持相关学校利用棚改腾空区拓展空间，改善师生学习生活环境。简化手续，加快进度，切实解决棚改回迁滞后的问题。持续加大城市老旧小区改造力度，再改造200余栋楼房，同步进行小区的绿化、美化、亮化。建设一批街区公园、袖珍花园、口袋广场，合理规划布局城区菜市场，方便群众生活，提高生活的舒适度。

　　四要着力完善社会保障体系。全面实施全民参保计划，持续扩大“五险”覆盖面，完善城镇居民医疗保险与新农合两项制度整合工作，实现跨省跨地区异地就医直接结算，推进参保人员网上缴费。完善最低生活保障制度，提高低保标准，推进低保人群医疗救助即时结算全覆盖，持续开展扶贫济困送温暖活动。大力发展养老事业，开展困境老人系列救助行动；探索启动居家养老照护服务，在社区建立老年人照料场所；支持养老产业发展，引导民间资本发展小微养老机构，在房、水、电、暖等方面给予优惠政策。鼓励有条件的县区利用闲置资源建设老年活动中心和老年大学；鼓励大型商场等公共场所设立敬老休闲区。

　　五要着力推动教育优先发展。像关心自己家孩子教育一样，重视教育工作，解决教育问题，促进教育事业发展。实施学前教育水平提升工程，建设高品质幼儿园。实施义务教育均衡发展工程，完成萝北县、绥滨县、兴安区、兴山区义务教育均衡发展工作。着力推动高中多元化特色化发展，建成16所特色学校。提高现代职业教育水平。加快智慧教育建设。有计划、分类别、全覆盖招聘中小学、幼儿教师，解决师资力量不足、音体美教师缺乏的问题。建设师德师风监督体系，对表现恶劣的教师一律清除出教师队伍；凡社会反响差的学校，对校长一律追责。高度重视校园安全，探索实施校办寄托模式，开办“阳光教室”，启动阳光志愿者活动，解决部分学生放学没人照管和吃饭家长不放心的问题。

　　六要着力保障人民群众健康。加快“健康鹤岗”建设。加大重点传染病、慢性非传染病防控力度，强化妇女儿童健康保障，加强弱势群体医疗救治。要提升医疗服务能力，全力抓好分级诊疗、家庭医生签约服务和医联体建设，建立以专家个人命名的“健康小屋”，让群众在家门口享受“专家级”服务。创建一所国家级优质服务示范社区卫生中心。大力发展“医养结合”新模式。推进卫生信息化建设。深化公立医院改革。强化医德医风监管，规范医疗服务行为。

　　七要着力加强公共文化服务。文化是城市之魂。要重视文化的培育和养成，推进文化进机关、进校园、进企业、进社区；安排、引导、支持开展多种形式的群众文化活动，培养扶持文艺团队及文艺骨干，每个月都要开展各类高品质文化活动，丰富百姓文化生活，提高文化素养和文化艺术鉴赏力。要完善文化基础设施，加快市县两级图书馆、艺术馆、文化馆达标建设，推进街道、社区、乡村文化场所建设，有计划建设一批反映地域特色文化的雕塑。要把文化建设融入文明城市创建当中，从不乱扔垃圾、汽车礼让行人、公共场所禁烟等身边的小事做起，着力提升居民的文明素质，在全社会营造热爱家乡、做文明市民、创文明城市的浓厚氛围。要出台政策，支持引导文化产业发展，利用闲置资产，为传播文化的团体协会提供支持。

　　八要着力发展群众体育事业。以群众需求为导向，大力推广和普及“四季”体育运动项目，加快培育具有鹤岗特色的群众性体育赛事活动，让百姓运动健身、关注参与赛事成为生活习惯。坚持“小型、多样、便捷”，完善群众身边的体育场地设施，支持有条件的区成立社区健身中心，打造“城市15分钟健身圈”。启动建设滑雪场、滑冰馆、体育公园。培育多元化体育市场主体，积极扶持各类体育协会、体育俱乐部发展壮大。强化社会体育指导员队伍建设，为群众提供全方位高质量的体育健身服务。

　　各位代表，人民对美好生活的向往就是我们的奋斗目标。我们要坚持把保障和改善民生作为一切工作的出发点和落脚点，努力在幼有所育、学有所教、业有可就、劳有所得、病有所医、老有所养、住有所居、弱有所扶上不断取得新进展。

　　(五)加强制度体系建设，提高社会治理水平

　　要提高社会治理社会化、法治化、智能化、专业化水平，打造共建共治共享的社会治理格局，让人民群众的安全感、获得感和幸福感得到进一步提升。

　　一要突出抓好安全生产工作。增强红线意识，开展“安全建设年”活动。严格落实安全生产责任制和管理、防范、监管、检查、奖惩措施。严厉打击违法违规生产行为，坚决用铁的手腕重点加强煤矿安全监管。全力抓好特种设备、交通、消防、非煤矿山、危险化学品、建筑行业和人员密集场所等重点领域的安全，坚决遏制重特大事故发生。要理顺食药监管职责，压实责任，抓好药品供应保障和医疗机构监管。实施食品安全战略，将更多的食品经营业户纳入“明厨亮灶”监管体系，提升百姓对食品安全的满意度。要提升综合防灾减灾能力，提高社会公众风险防范意识和自救互救技能。

　　二要突出抓好群众信访工作。要把信访工作作为了解民情、集中民智、维护民利、凝聚民心的一项重要工作，千方百计为群众排忧解难，真正把解决信访问题的过程作为践行党的群众路线、做好群众工作的过程。坚持“阳光信访”，紧扣“事要解决”这个核心，切实依法及时就地解决群众合理诉求，带着感情和责任维护群众合法权益。坚持“责任信访”，执行好信访接待日、领导包案、首问责任制等工作制度。坚持“法治信访”，依法处理无理缠访、闹访等行为，依法打击信访活动中违法犯罪行为。

　　三要突出抓好社会治安工作。提高社会治安防控信息化、精准化水平，落实社会治安打、防、控措施，严打暴恐犯罪，坚决做到黄赌毒必治、黑拐骗必除，实施“满天星”工程，打造“平安之城”，切实保护好人民群众的人身权、财产权和人格权。强化社会治安保障，完善各类预案，提升应急处置能力。在医院、学校、车站、商场等人员密集的重点场所，建立完善派驻警力或专业安保人员机制，提升人民群众的安全感。

　　四要突出抓好诚信建设工作。建立以政务诚信、商务诚信、社会诚信和司法诚信建设为主要内容的诚信格局。加快建立失信惩戒和守信激励机制，完善各领域“黑名单”制度，重点将安全生产“胆大妄为者”、欠债不还的“老赖”、食品药品经营“黑心人”列入黑名单当中，实行联合惩戒。广泛开展诚信行业、诚信单位、诚信窗口主题实践活动，发掘树立典型，弘扬诚信精神。

　　五要突出抓好城市管理工作。推动城建法规制定，夯实规划、建设、管理三大环节保障支撑。深化城市专项治理，开展环境卫生、临街牌匾规范等专项整治行动；实现“弃管楼”物业管理全覆盖，加强行业监管，实施企业清退制度；统一规范道路、小区名称，丰富文化内涵，增强城市归属感；就近就便建设移动式环卫工人临时休息房。规范建筑市场，全面提升建筑工程安全质量，妥善处置“烂尾楼”工程。从严整治违建，坚决做到新增违章建筑“零容忍”，强化拆违后土地的绿化、硬化、美化，切实做到还地于民、还利于民。每月召开一次民情恳谈会，建立供水、供电、供热等重点民生工作快速回应机制，开设“鹤岗民情”网站，畅通与市民联系沟通渠道。严厉打击“黑车”和超限超载等违法行为。加强学校、医院等重点地段交通管理工作。要规范执法行为，先热情服务，后严格执法。优化调整全市公交线路，方便百姓出行。

　　六要突出抓好社区治理工作。加快动迁居民户籍调整工作，解决群众办事不方便的问题。加强硬件建设，新建社区服务中心面积都要达到1000平方米以上，合理规划建设文化、体育、商业、快递等自助服务设施，为居民提供家门口的服务。推进学习型和智慧社区建设，实施“互联网+社区”行动计划。要建立社区工作事项准入制度，为社区减负。开展“两清理”行动，全面清理规范各单位要求社区出具的各类证明，全面清理政府部门在社区设立的工作机构和加挂的各种牌子，精简社区各类会议和工作台账，让社区干部腾出时间和精力更好地为群众服务。

　　各位代表，我们要通过不断提高新时代新时期下的社会治理水平，实现政府治理和社会调节、居民自治良性互动，让每一个在鹤岗生活、创业的人平安、平和、平等！

　　三、强化保障措施，增强经济社会发展动力

　　(一)支持实体经济发展。开展工业企业提质增效专项行动，实施“规上工业保卫工程”，对临规企业实行联合帮扶和靶向培育，新增规模以上工业企业10户。对存在困难的企业，一企一策，帮助企业解决重点难点问题，确保生产企业发挥最大效益。对停产半停产企业，分类施策，淘汰一部分，激活一部分，加大引联力度，推动闲置的存量转化为新的增量。

　　(二)实施精准招商。要以招商引资促进产业发展。要厘清优势劣势，明确招商方向，出台投资优惠政策，建立健全招商激励机制，提高招商实效。瞄准广东等发达地区定向招商。市级领导要带头招商，县区要发挥招商主体作用，在全市掀起招商热潮。要高度重视和支持本土企业发展，引导本土企业家再创业，引导走出去的鹤岗籍企业家回家创业。

　　(三)推进园区建设。落实鹤岗市经济开发区总体规划、煤化工园区产业发展规划，探索制定入驻园区项目承诺制服务办法，为项目引进打好基础。强化园区基础设施建设，重点推进煤化工园区铁路专用线及园区道路工程，谋划建设绿色食品产业园区、煤化工园区污水处理厂。加强市、县两级石墨园区水、电、气、热、通讯、道路、污水处理、大型尾矿库等基础设施建设，为项目落地提供要素保障。加快园区土地收储，依法依规清理闲置低效土地。

　　(四)推动民营经济“大发展”。出台有鹤岗特点的支持民营经济发展的意见，整合民营经济的工作力量，建立专项领导小组，成立民营经济促进局，在市场准入、市场开拓、品牌扶持、上市扶持资金等方面给予企业政策扶持。全面建立中小企业商会、在鹤投资的外地企业商会和在外地投资的鹤岗企业商会，并发挥好作用。用好国家和省促进市场主体发展的各项政策，支持引导中小微企业、个体工商户的发展。加强优秀企业家培育。实施好金融扶持政策。

　　(五)加快区域合作。支持农垦、森工、龙煤的改革，找准结合点，生产要素统筹配置、资源共享，推进区域联合招商、联合上项目，促进融合发展。深入实施“开放活市”战略，推进“外放内活”，发展飞地经济。制定口岸建设规划，健全互联互通设施，扩大与俄罗斯等国家和地区的经贸往来和文化交流。抓住鹤岗、汕头对口合作机遇，探索建立跨区域利益分享机制，全面推进两市多领域深入合作。

　　(六)打好“互联网+双创”融合提升攻坚战。坚持鼓励支持和规范发展并行，政策引导与依法管理并举，经济效益和社会效益并重，深入实施全市“互联网+”行动计划，积极稳妥推进我市移动互联网健康有序发展。充分发挥“双创”基地、孵化器航母的引领带动作用，推进我市企业品牌孵化，促进孵化企业提档升级。市县区联动建立科技、人才、投资等各具特色的孵化器，促进创业创新资源向孵化领域加速集聚。加强产学研合作，持续开展“金鹤爱岗”科技成果转化活动。要为各类人才发挥作用提供平台和渠道，聚人才、兴事业。

　　(七)打好重要领域改革攻坚战。要用改革创新激发城市活力动力潜力，承接好中央、省改革任务，坚持问题导向，刀刃向内，推动自我改革创新。行政机关、事业单位改革要有大突破，解决“有事没人干、有人没事干”的问题。要引导鼓励机关事业单位人员从体制内走出，外出创业。要全面启动28家生产经营类事业单位改革，割断事业身份脐带，让企业真正走向市场。鼓励社会资本参与事业单位改革。要以产业发展引领推动林业、农业、粮食、供销系统公司化改革；要以产业发展推进文化、旅游、体育、医疗、养老等领域的改革，组建林业、粮食、文旅、广电传媒四大集团。以这些单位的改革为示范牵动，推动其他事业单位改革。

　　(八)打好防范化解重大风险攻坚战。有效控制政府债务。建立健全风险预警和应急处置机制，努力控制政府债务增长。强化金融监管，严厉打击非法集资等犯罪活动，严格规范民间借贷，坚决取缔非法金融机构，坚决守住风险防范底线，防止经济金融风险演化为政治社会风险。

　　(九)打好整顿作风优化发展环境攻坚战。良性发展需要有良好的环境来支持。对鹤岗而言，环境优势比资源优势更重要，工作作风比工作能力更重要。要按照打造全省最优发展环境的目标，确立工作标准、完善工作机制、改进工作作风、整改工作问题、提高工作质量。坚持治标与治本结合，优化与惩治并重，向企业最急需、最关心的问题发力，向群众最不满意的事发力。发挥“企业投诉平台”作用，规范涉企检查，确保企业正常生产经营“零干扰”。建立与企业家沟通联系机制，每月召开一次行业企业家座谈会，经常性对企业开展调研，及时解决反映强烈的突出问题。优化“流程再造”，达到“流程最优、环节最少、时间最短、服务最佳”的目标。推行政务服务事项网上办理，方便人民群众办事。对破坏发展环境的人和事“零容忍”，严厉查处不作为、乱作为、慢作为等破坏发展环境的行为。

　　各位代表，我们要完善制度机制，强化保障措施，营造良好经济社会生态，让所有价值创造者在鹤岗这片土地上安心投资、舒心发展！

　　四、加强政府自身建设，展现新作为、再创新业绩

　　2018年的工作任务已经明确，相信这些任务的顺利完成，一定会给鹤岗带来新的更大变化。习总书记告诉我们，“社会主义是干出来的”！重担已经摆在我们每个人的面前，我们不能有丝毫的犹豫和懈怠，要继续保持旺盛的斗志、冲天的干劲，忠于职守，攻坚克难，任劳任怨，苦干实干，不断提高政府行政水平。

　　——要履职尽责弘扬正气。要形成严格自律好作风，真正把纪律和规矩立起来、严起来，严格按照法定权限和程序行使权力、履行职责。要坚持用制度管权、管事、管人，用制度培养习惯，用程序规范行为，改变法律意识淡薄、制度执行不力的状况，杜绝违纪违规行为的发生，建设风清气正的良好政治生态，树立廉洁干净的政府形象。

　　——要俯下身子真干实事。要形成马上就办的作风，争做“雷厉风行的快把式”、“埋头苦干的真把式”，坚决向“表态多调门高，行动少落实差”的行为说不，让抓落实成为政府工作的“基本功”和“主旋律”。要对作出的承诺、立下的军令状敲钟问响，说一件、干一件、成一件，用弯下的腰、流下的汗在群众心中树起挺直的风骨。

　　——要敢于革新勇挑重担。要敢闯敢试、敢于求变，勇于借鉴外地先进经验，对标发达地区大胆试验，推动鹤岗各项工作上水平。要敢于碰矛盾、碰问题，坚决摒弃“只求不出事，宁愿不做事”的态度，铸就勇于挑担的“铁肩膀”。形成“头雁效应”，一级做给一级看、一级带着一级干，带动全市革故鼎新、干事创业。

　　——要追求卓越争创一流。要形成勤于学习、严谨细致、勤勉高效好作风，用新理念新方法引领工作。坚决摒弃“走秀式”调研方式，充分尊重、吸纳各方意见，坚持工作在一线，在一线发现问题、解决问题，争做“追求卓越的好把式”。增强创先意识，提高工作标准，勇于超越，在一些关键领域、重点工作上走在全省前列。

　　新时代、新征程、新任务，对政府自身建设提出了新的更高要求。我们要全面深入学习习近平新时代中国特色社会主义思想，党的十九大精神和省市委全会精神，聚焦习近平总书记指出的10个方面形式主义、官僚主义具体表现，下大力气加以整治，驰而不息改进作风。要牢牢把握正确的工作方向、工作理念，增强“四个意识”，树牢“以人民为中心”的发展思想，遵循开拓、务实、高效、廉洁的原则，切实增强“八个本领”，以全新境界和超常措施，建设让人民满意的法治政府、创新政府、廉洁政府和服务型政府。

　　各位代表，在一个城市发展的历程中，一年只是短暂的瞬间，即使耗尽心血、穷尽才力也未必能够成就辉煌与伟业。但是一天一天的积累、一月一月的奋斗，却可以为梦想奠基、为后人筑路，这也是我们必须担当的责任与使命。我们坚信，在省委、省政府和市委的正确领导下，经过全市人民的共同努力，我们“加速转型发展、决胜全面小康，建设富强民主文明和谐美丽的社会主义现代化新鹤岗”的美好蓝图一定能够早日实
